# Supplementary material for: Multiplexed imaging analysis of the tumor-immune microenvironment reveals predictors of outcome in triple-negative breast cancer
Source: Commun Biol. 2021 Jul 9;4:852. doi: 10.1038/s42003-021-02361-1 (PMC8271023; doi:10.1038/s42003-021-02361-1)
Supplement: Supplementary file 1 — Supplementary Information [file 42003_2021_2361_MOESM1_ESM.pdf]

Multiplexed Imaging Analysis of the Tumor-Immune Microenvironment Reveals Predictors of Outcome in Triple-Negative Breast Cancer

Aalok Patwa[1, 2*], Rikiya Yamashita[1,3*], Jin Long[3], Tyler Risom[4], Michael Angelo[4], Leeat Keren[5], Daniel L. Rubin[1,3**]

1. Department of Biomedical Data Science, Stanford University, Stanford, CA 94305, USA
2. Archbishop Mitty High School, San Jose, CA 95129, USA
3. Center for Artificial Intelligence in Medicine and Imaging, Stanford University, Stanford, CA 94305, USA
4. Department of Pathology, Stanford University, Stanford, CA 94305, USA
5. Department of Molecular Cell Biology, Weizmann Institute of Science, Rehovot, Israel

* These authors contributed equally.
** Corresponding author: dlrubin@stanford.edu

Supplementary Information

**Functional Proteins**	Beta Catenin, CD138, CD45RO, CD63, FoxP3, H3K27me3, H3K9ac, HLA-DR, HLA Class 1, IDO, Keratin17, Keratin6, Ki67, Lag3, p53, PD-L1, PD-1, Phospho-S6
**Lineage Proteins**	CD11b, CD11c, CD16, CD20, CD209, CD3, CD31, CD4, CD45, CD56, CD68, CD8, dsDNA, EGFR, MPO, Pan-Keratin, SMA, Vimentin

Supplementary Table 1. Delineation between functional and lineage proteins. Lineage proteins are those that are solely used for the identification of cell type, whereas functional proteins provide information related to proliferation or metabolic activity[25].

Protein	Coefficient	Hazard Ratio	Coefficient P	BH-Corrected FDR
**CD45RO**	-0.019	0.981	0.051	0.311
**PD1**	-0.112	0.894	0.063	0.311
**CD138**	0.018	1.018	0.069	0.311
**IDO**	-0.067	0.935	0.084	0.311
**HLA_Class_1**	-0.053	0.948	0.104	0.311
**HLA-DR**	-0.013	0.987	0.12	0.311
**Lag3**	-0.232	0.793	0.121	0.311
**p53**	-0.023	0.978	0.212	0.477
**H3K9ac**	0.086	1.09	0.331	0.661
**H3K27me3**	0.724	2.063	0.367	0.661
**Keratin17**	0.009	1.009	0.432	0.684
**Beta catenin**	-0.008	0.992	0.476	0.684
**phospho-S6**	0.012	1.012	0.494	0.684
**Keratin6**	0.005	1.005	0.651	0.837
**PD-L1**	-0.004	0.996	0.715	0.858
**CD63**	0.003	1.003	0.791	0.890
**Ki67**	-0.007	0.993	0.846	0.892
**FoxP3**	-0.027	0.973	0.892	0.892

Supplementary Table 2. Protein expression Cox regression results for recurrence. This table shows the results from performing univariate Cox regression with each protein's expression as predictors and recurrence outcome as the response on the 38 TNBC images. There is no association between protein expression and recurrence in the cohort.

Protein	Coefficient	Hazard Ratio	Coefficient P	BH-Corrected FDR
**Keratin6**	0.025	1.025	0.034	0.374
**HLA-DR**	-0.018	0.982	0.045	0.374
**Lag3**	-0.336	0.715	0.073	0.374
**Keratin17**	0.02	1.021	0.096	0.374
**HLA_Class_1**	-0.055	0.946	0.109	0.374
**H3K27me3**	-0.073	0.93	0.146	0.374
**IDO**	-0.049	0.952	0.164	0.374
**CD138**	0.014	1.014	0.175	0.374
**PD1**	-0.069	0.934	0.187	0.374
**CD45RO**	-0.011	0.989	0.241	0.434
**p53**	-0.014	0.987	0.353	0.578
**CD63**	0.008	1.008	0.511	0.671
**Beta catenin**	-0.007	0.993	0.517	0.671
**H3K9ac**	0.056	1.058	0.522	0.671
**PD-L1**	-0.006	0.994	0.626	0.751

**FoxP3**	-0.085	0.918	0.688	0.774
**Ki67**	0.009	1.009	0.771	0.816
**phospho-S6**	0.001	1.001	0.928	0.928

Supplementary Table 3. Protein expression Cox regression results for survival. This table shows the results from performing univariate Cox regression with each protein's expression as predictors and survival outcome as the response on the 38 TNBC images. There is no association between protein expression and survival in the cohort.

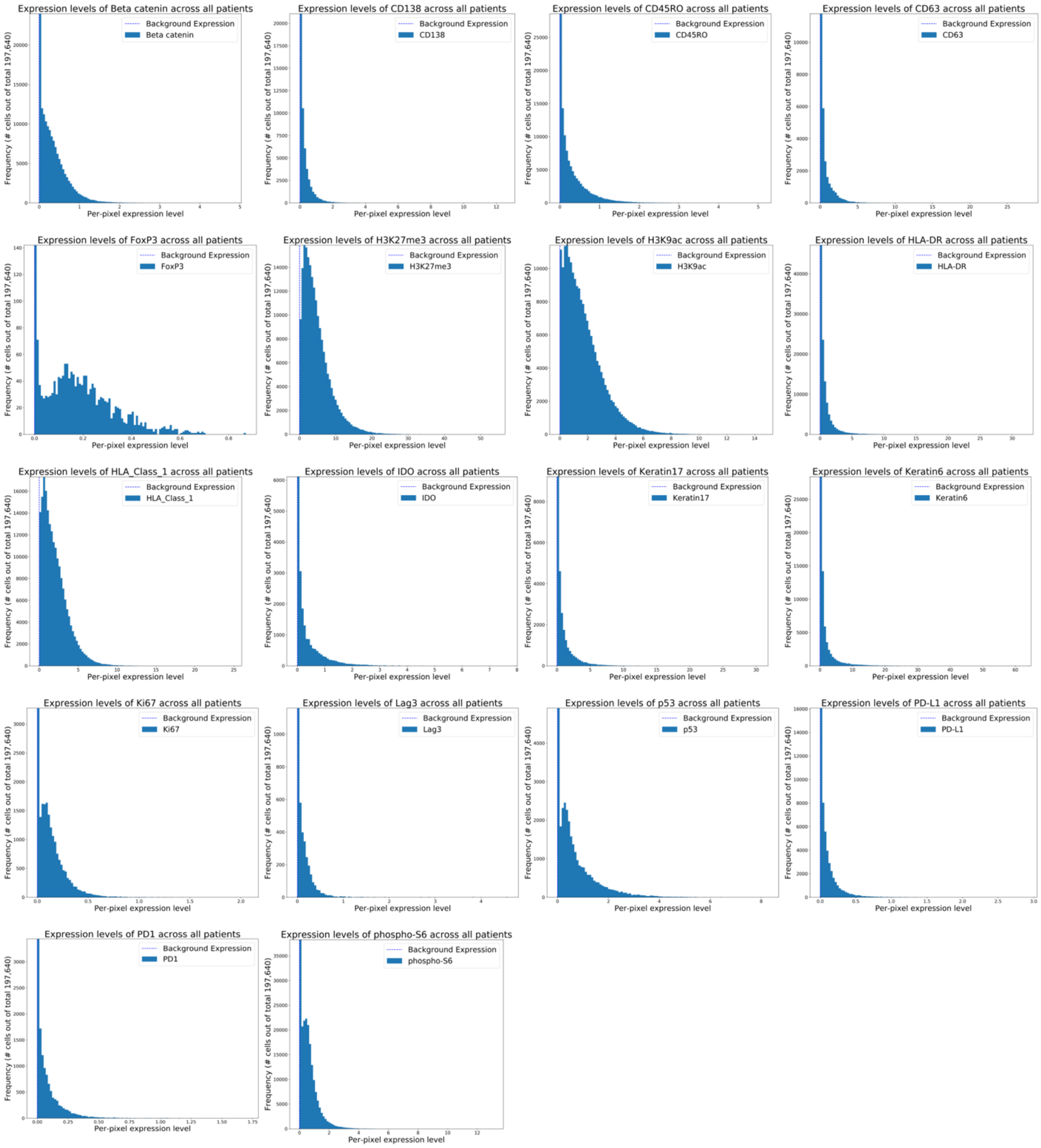

Supplementary Figure 1. Histograms of expression for all functional proteins. Each of the plots shown in the figure is a histogram of the per-pixel expression levels of that protein across all of the cells in all of the patients. The y-axis has been shrunk to better show the distribution, as in reality, the values are extremely right-skewed, and the frequency of 0 expression dwarfs the rest of the values. However, it is still valuable to see the rest of the distribution, as this is where the difference in cellular phenotype is made. As such, the y-axis upper limit has been made twice the second highest frequency.

Features	Chosen Clusters	Mean Silhouette Score
Protein co-expression	2	0.42
	3	0.18
	4	0.04
	5	0.04
	6	0.02
Functional proteins interactions	2	0.38
	3	0.11
	4	0.05
	5	0.04
	6	-0.23
Immunoregulatory proteins interactions	2	0.47
	3	0.17
	4	0.06
	5	0.04
	6	-0.29

Supplementary Table 4. Silhouette score analysis on hierarchical clustering. This table shows the mean silhouette score of hierarchical clustering based on the number of chosen clusters for each spatial analysis. In all cases, choosing two clusters led to the highest silhouette score.

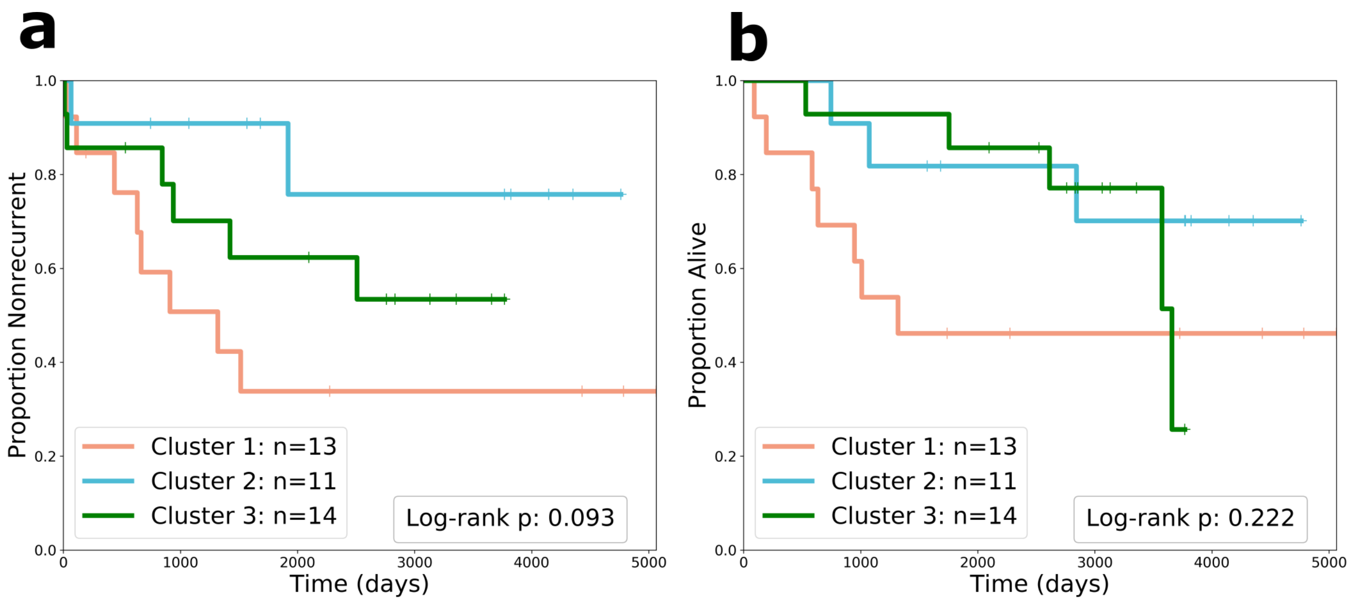

Supplementary Figure 2. Kaplan-Meier curves comparing three patient clusters chosen from protein co-expression features. a Kaplan-Meier curve comparing recurrence across the three patient clusters. The two-sided log-rank test (df=2) p-value is shown. **b** Kaplan-Meier curve comparing survival across the three patient clusters. The two-sided log-rank test (df=2) p-value is shown.

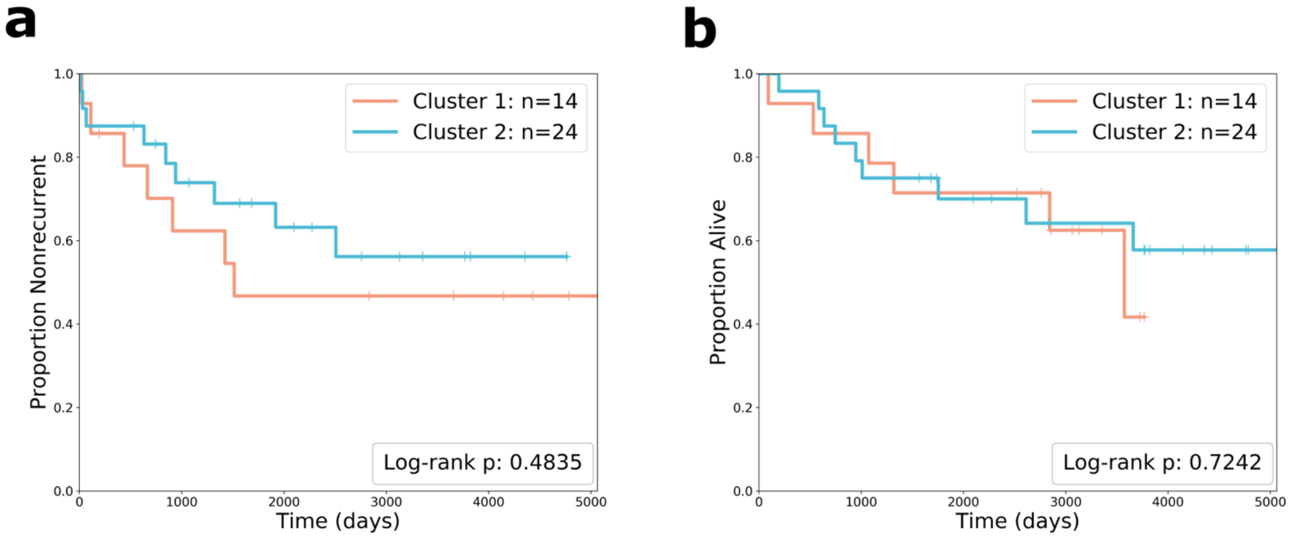

Supplementary Figure 3. Kaplan-Meier curves comparing two patients clusters chosen from lineage proteins interactions features. **a** Kaplan-Meier curve comparing recurrence outcomes across patient clusters formed from lineage proteins interactions features. There is not clear divergence between the clusters. The two-sided log-rank test (df=1) p-value is shown. **b** Kaplan-Meier curve comparing overall survival across patient clusters formed from lineage proteins interactions features. There is no clear divergence.

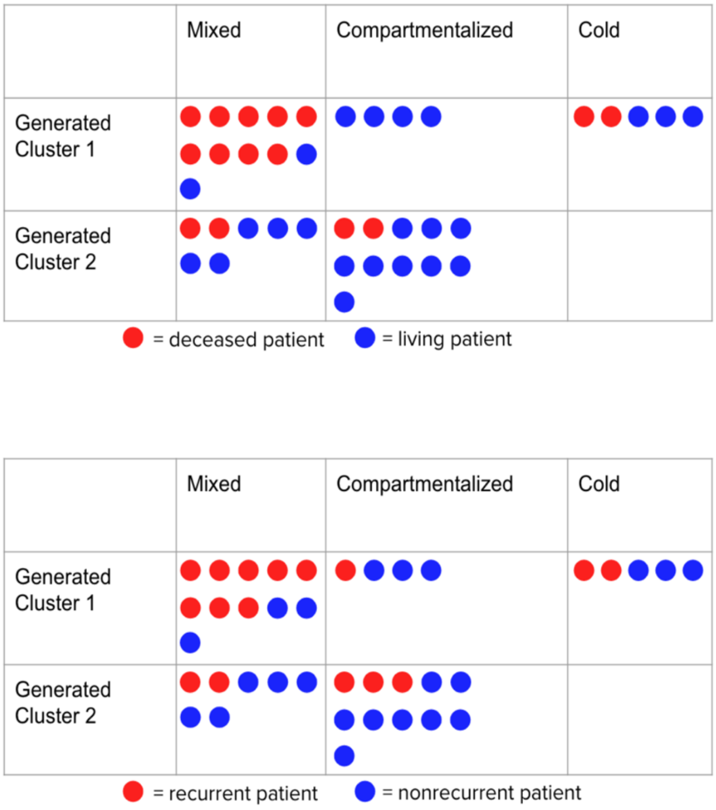

Supplementary Figure 4. Drawing comparing the patients in clusters formed from interaction features to Keren et al.'s designation of "mixed", "compartmentalized", and "cold" architectures.

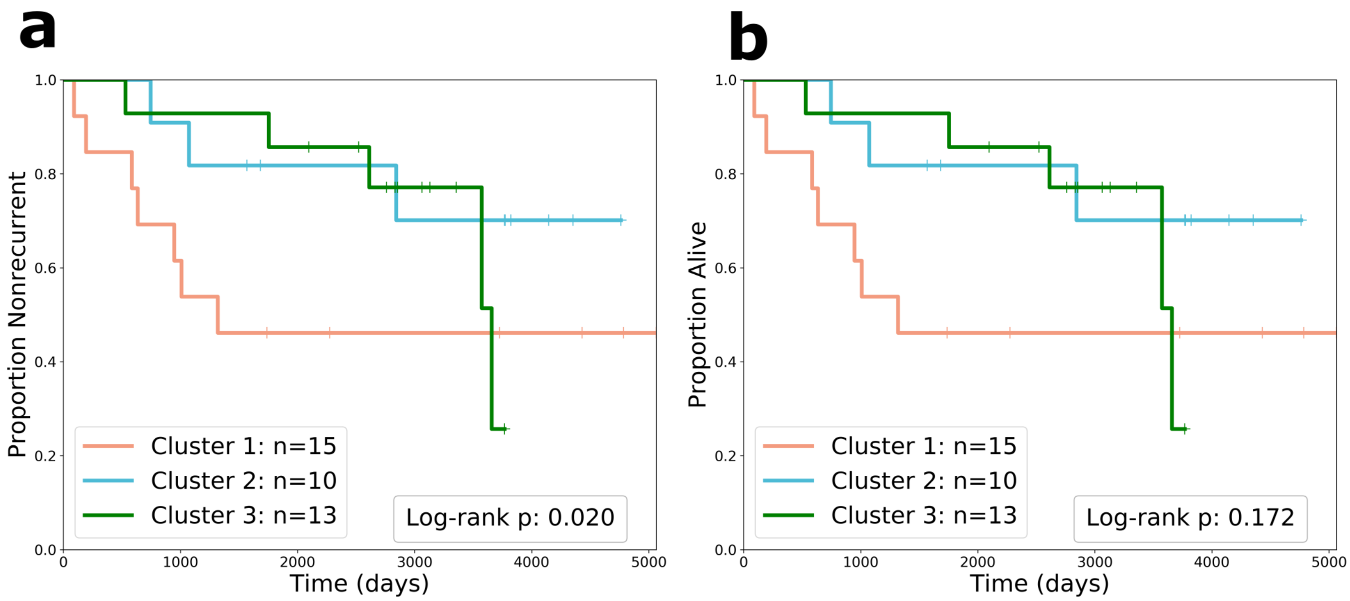

Supplementary Figure 5. Kaplan-Meier curves comparing three patient clusters chosen from immunoregulatory protein interaction features. a Kaplan-Meier curve comparing recurrence outcomes across the three patient clusters. The two-sided log-rank test (df=2) p-value is shown. **b a** Kaplan-Meier curve comparing survival across the three patient clusters. The two-sided log-rank test (df=2) p-value is shown.

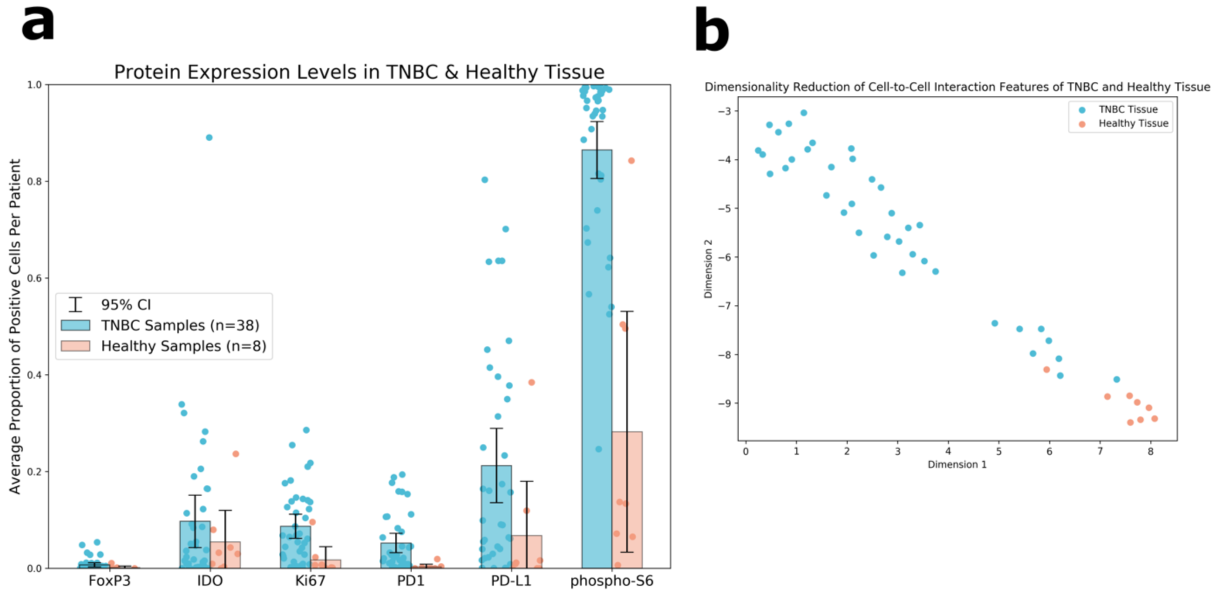

Supplementary Figure 6. Healthy tissue analysis. a Double bar plot comparing the expression levels of six proteins between TNBC patients' images (n=38) and healthy patients' images (n=8). The error bars show the 95% confidence interval. **b** Scatterplot showing cell-to-cell interaction features reduced to two dimensions using UMAP. The healthy samples are bunched together.

Algorithm 1 RASP-MIBI Computational Pipeline

```
input: image cohort of size N, segmentations of T cell types, MIBI images
for K markers.

# Immune Composition Analysis
for cell type {c_t}_{t=1}^T do
    for patient segmentation {s_n}_{n=1}^N do
        numTotal ← total cells in s_n
        numCell ← number of cells of type c_t in s_n
        prevCell ← numCell / numTotal
    end for
    tTest(recurrence, prevCell)
    tTest(survival, prevCell)
end for

# Protein Expression Analysis
for marker {m_k}_{k=1}^K do
    backgroundPixels ← total pixels of slide background across all patients
    backgroundExp ← Σ_{pixel=1}^{backgroundPixels} expression of m_k in pixel
    perPixelThreshold ← backgroundExp / backgroundPixels
    for patient MIBI image {p_n}_{n=1}^N do
        numTotal ← total cells in p_n
        numPositive ← 0
        for cell {C_j}_{j=1}^{numTotal} do
            numPixels ← number of pixels in C_j
            exp ← Σ_{pixel=1}^{numPixels} expression of m_k in pixel
            perPixelExp ← exp / numPixels
            positivityBin ← perPixelExp > perPixelThreshold
            if positivityBin then
                numPositive += 1
            end if
        end for
        proportionPositive ← numPositive / numTotal
    end for
    tTest(recurrence, proportionPositive)
    tTest(survival, proportionPositive)
end for

# Protein Co-Expression Analysis
for patient MIBI image {p_n}_{n=1}^N do
    coexpMatrix ← matrix of co-expressions of protein pairs across all cells
end for
hierarchicalClustering(patients, coexpMatrix)
logrankTest(cluster1, cluster2, recurrence)
logrankTest(cluster1, cluster2, survival)
```

```
# Voronoi Tessellation
for patient MIBI image {p_n}_{n=1}^N do
    for marker {m_a}_{a=1}^K do
        for marker {m_b}_{b=1}^K do
            interactionMatrix[m_a, m_b] ← 0
        end for
    end for
    V ← drawVoronoiDiagram
    for edge in V do
        cell_p ← first cell in edge
        cell_q ← second cell in edge
        for (m_a, m_b) in interactionMatrix do
            if (cell_p positive for m_a AND cell_q positive for m_b) OR (cell_p positive
            for m_b AND cell_q positive for m_a) then
                interactionMatrix[m_a, m_b] += 1
            end if
        end for
    end for
    return interactionMatrix
end for

# Interaction Analysis
hierarchicalClustering(patients, interactionMatrix)
logrankTest(cluster1, cluster2, recurrence)
logrankTest(cluster1, cluster2, survival)
```

Supplementary Figure 7. Pseudocode for the main computational pipeline. The major steps in the analysis pipeline are shown. Details regarding algorithms and techniques used are provided.
