# Supplementary material for: Multiplexed imaging analysis of the tumor-immune microenvironment reveals predictors of outcome in triple-negative breast cancer
Source: Commun Biol. 2021 Jul 9;4:852. doi: 10.1038/s42003-021-02361-1 (PMC8271023; doi:10.1038/s42003-021-02361-1)
Supplement: Supplementary file 2 — Reporting Summary [file 42003_2021_2361_MOESM2_ESM.pdf]

nature research

Corresponding author(s): Daniel Rubin

Last updated by author(s): May 29, 2021

Reporting Summary

Nature Research wishes to improve the reproducibility of the work that we publish. This form provides structure for consistency and transparency in reporting. For further information on Nature Research policies, see our Editorial Policies and the Editorial Policy Checklist.

Statistics

For all statistical analyses, confirm that the following items are present in the figure legend, table legend, main text, or Methods section.

n/a	Confirmed	
☐	☒	The exact sample size (*n*) for each experimental group/condition, given as a discrete number and unit of measurement
☐	☒	A statement on whether measurements were taken from distinct samples or whether the same sample was measured repeatedly
☐	☒	The statistical test(s) used AND whether they are one- or two-sided<br>*Only common tests should be described solely by name; describe more complex techniques in the Methods section.*
☐	☒	A description of all covariates tested
☐	☒	A description of any assumptions or corrections, such as tests of normality and adjustment for multiple comparisons
☐	☒	A full description of the statistical parameters including central tendency (e.g. means) or other basic estimates (e.g. regression coefficient) AND variation (e.g. standard deviation) or associated estimates of uncertainty (e.g. confidence intervals)
☐	☒	For null hypothesis testing, the test statistic (e.g. F, t, r) with confidence intervals, effect sizes, degrees of freedom and P value noted<br>*Give P values as exact values whenever suitable.*
☒	☐	For Bayesian analysis, information on the choice of priors and Markov chain Monte Carlo settings
☒	☐	For hierarchical and complex designs, identification of the appropriate level for tests and full reporting of outcomes
☒	☐	Estimates of effect sizes (e.g. Cohen's d, Pearson's r), indicating how they were calculated

Our web collection on statistics for biologists contains articles on many of the points above.

Software and code

Policy information about availability of computer code

Data collection	TNBC patients' data was obtained from a previous study by Keren et al., who made their data publicly available. Healthy patients' data was obtained by a previous study by Risom et al. In these studies, H&E stained samples of breast tissue were scanned used MIBI-TOF. DeepCell was used to segment and classify cells in the images.
Data analysis	All data analysis was performed using Python (3.7.3) and code for analysis can be found at github.com/aalokpatwa/rasp-mibi.

For manuscripts utilizing custom algorithms or software that are central to the research but not yet described in published literature, software must be made available to editors and reviewers. We strongly encourage code deposition in a community repository (e.g. GitHub). See the Nature Research guidelines for submitting code & software for further information.

Data

Policy information about availability of data

All manuscripts must include a data availability statement. This statement should provide the following information, where applicable:

- Accession codes, unique identifiers, or web links for publicly available datasets
- A list of figures that have associated raw data
- A description of any restrictions on data availability

MIBI images and other raw data for TNBC patients can be found at https://mibi-share.ionpath.com/. The link comes with an easy-to-use interface that allows for easy examination of the data upon registration. MIBI images for healthy patients will soon be made available on a Human Tumor Atlas Network public repository. The data produced by intermediary steps in the computational pipeline can be found at github.com/aalokpatwa/rasp-mibi/ in the intermediate_data/ folder.

Field-specific reporting

Please select the one below that is the best fit for your research. If you are not sure, read the appropriate sections before making your selection.

[x] Life sciences [ ] Behavioural & social sciences [ ] Ecological, evolutionary & environmental sciences

For a reference copy of the document with all sections, see nature.com/documents/nr-reporting-summary-flat.pdf

Life sciences study design

All studies must disclose on these points even when the disclosure is negative.

Sample size	Sample size was chosen based on the availability of MIBI images of triple-negative breast cancer patients and healthy patients. Our sample size was sufficient for our study because of its exploratory nature, as well as our random survival forest's strong performance on a held-out validation set.
Data exclusions	Three TNBC patients were excluded from the original dataset. Two of them did not have recorded recurrence outcomes, making them unsuitable for our analysis. The third patient had a corrupted image.
Replication	For hierarchical clustering, multiple linkage methods and dissimilarity metrics were tested. For the random survival forest analysis, the performance of the model was evaluated using a held-out validation set.
Randomization	Images were assigned to the validation set randomly.
Blinding	Observers were blinded to clinical data during data collection. However, blinding was not possible for our analysis because we examined the differences in clinical outcome across patient clusters formed from biological features. Our purpose was to investigate the clinical data.

Reporting for specific materials, systems and methods

We require information from authors about some types of materials, experimental systems and methods used in many studies. Here, indicate whether each material, system or method listed is relevant to your study. If you are not sure if a list item applies to your research, read the appropriate section before selecting a response.

Materials & experimental systems

n/a	Involved in the study	
[x]	[ ]	Antibodies
[x]	[ ]	Eukaryotic cell lines
[x]	[ ]	Palaeontology and archaeology
[x]	[ ]	Animals and other organisms
[ ]	[x]	Human research participants
[x]	[ ]	Clinical data
[x]	[ ]	Dual use research of concern

Methods

n/a	Involved in the study	
[x]	[ ]	ChIP-seq
[x]	[ ]	Flow cytometry
[x]	[ ]	MRI-based neuroimaging

Human research participants

Policy information about studies involving human research participants

Population characteristics	All relevant population characteristics are reported in the manuscript in Table 1.
Recruitment	The authors had no role in the recruitment of participants. Patients were chosen by previous studies.
Ethics oversight	Stanford University Institutional Review Board

Note that full information on the approval of the study protocol must also be provided in the manuscript.
